# Serum uric acid is independently and linearly associated with risk of nonalcoholic fatty liver disease in obese Chinese adults

**DOI:** 10.1038/srep38605

**Published:** 2016-12-07

**Authors:** Chang-Qin Liu, Chun-Mei He, Ning Chen, Dongmei Wang, Xiulin Shi, Yongwen Liu, Xin Zeng, Bing Yan, Suhuan Liu, Shuyu Yang, Xiaoying Li, Xuejun Li, Zhibin Li

**Affiliations:** 1 Department of Endocrinology and Diabetes, the First Affiliated Hospital, Xiamen University, Xiamen, China; 2 Xiamen Diabetes Institute, Xiamen, China; 3 School of Public Health, Xiamen University, Xiamen, China; 4 Department of Endocrinology, Zhongshan Hospital, Fudan University, Shanghai, China; 5 Epidemiology Research Unit, the First Affiliated Hospital, Xiamen University, Xiamen, China

## Abstract

The present study aimed to explore the independent association and potential pathways between serum uric acid (SUA) and nonalcoholic fatty liver disease (NAFLD). 1365 community-living obese Chinese adults who received hepatic ultrasonography scanning were included. The prevalence rates of NAFLD were 71.5% for men and 53.8% for women. Compared with controls, NAFLD subjects showed significantly increased SUA levels (333.3 ± 84.9 v.s. 383.4 ± 93.7 μmol/L) and prevalence rate of hyperuricemia (HUA) (25.7% v.s. 47.3%, p < 0.001). After adjustment for insulin resistance (IR), components of metabolic syndrome (MetS) and other potential confounders, elevated SUA is independently associated with increased risk of NAFLD, with the adjusted OR of 1.528–2.031 (p < 0.001). By using multivariable fractional polynomial (MFP) modeling, the best FP transformation model shows that SUA was independently and linearly associated with risk of NAFLD. The one-pathway model by using structural equation modeling (SEM) about the relationships among SUA, IR, components of metabolic syndrome and NAFLD fits well (χ^2^ = 57.367, p < 0.001; CFI = 0.998; TLI = 0.992; and RMSEA = 0.048) and shows SUA might increase the risk of NAFLD directly besides of the indirect effects through increasing fasting insulin, blood pressure, triglyceride and decreasing HDL-C levels. Our results imply that elevated SUA may play an important role in NAFLD pathogenesis.

Nonalcoholic fatty liver disease (NAFLD), typically comprises a spectrum of pathological conditions including simple steatosis, nonalcoholic steatohepatisis (NASH) and cirrhosis due to significant fat accumulation in the liver, is becoming a challenge for public health because of its increasing prevalence worldwide and its consequences not only for chronic liver disease and hepatocellular carcinoma but also for extra-hepatic diseases, such as type 2 diabetes mellitus (T2DM), cardiovascular disease (CVD), and chronic kidney disease^1^,^2^,^3^,^4^. For general populations, the prevalence rate of NAFLD is estimated to be approximately 20–30% in Western countries and 5–18% in Asia^2^. Understanding the risk factors of NAFLD and identifying individuals with specific risk factors remain important prevention strategies due to no proved intervention for treatment of NAFLD.

SUA has been consistently shown to be associated with hypertension, insulin resistance, abdominal obesity and dyslipidemia, a cluster of metabolic disorders that is identified as metabolic syndrome (MetS)^5^,^6^. Since NAFLD has also been regarded as a hepatic manifestation of MetS, growing interests have focused on the relationship between SUA and NAFLD. A few evidences have documented that elevated SUA is associated with development or progression of NAFLD for eastern Asian populations^7^,^8^,^9^. Recent studies of the Third National Health and Nutrition Examination Survey (NHANES III) (1988–1994) also found that hyperuricemia (HUA) or increased quartiles of SUA was associated with increased risk of NAFLD in the US populations^10^,^11^. Whether the association between SUA and risk of NAFLD is independent of insulin resistance and components of MetS is still unknown due to the complex interrelationships among them. Furthermore, there is no evidence available on the possible pathways among SUA, insulin resistance, components of MetS and NAFLD, therefore studies are warranted to address this discrepancy, which may be helpful in elucidating the mechanisms of NAFLD.

An important issue in identifying possible risk factors is the nature of the relationship between the covariates and the dependent variables. Continuous covariates are often simplified by assuming a linear relationship or by categorization (frequently dichotomization) in the multivariable regression analysis. Such a modeling strategy has often been criticized in the statistical literature^12^. Sauerbrei *et al*. proposed the multivariable fractional polynomial (MFP) modeling as a systematic approach to investigate possible non-linear relationships in the multivariable regression analysis^13^,^14^. As for analysis of association between SUA and NAFLD, all of the available evidence regarded SUA as either continuous variable by assuming a linear relationship or categorical variable (quartiles, quintiles or dichotomization into HUA), which may result in considerable loss of power and residual confounding. Therefore, exploring whether there is a non-linear relationship between SUA and NAFLD, especially for values of SUA within the normal range, using MFP modeling approach is needed.

In the present study of 1365 community-living healthy obese Chinese adults with the same baseline examination as that of our previous publications^15^,^16^, we firstly aimed to examine the association of SUA with NAFLD, independent of insulin resistance and components of MetS. Secondly, we aimed to test if there is a non-linear relationship between SUA level and risk of NAFLD by using MFP modeling. Thirdly, we will explore the possible pathways among SUA, insulin resistance, components of MetS and NAFLD.

## Results

Of the 1365 participants, 944 (69.2%) were female. The mean age (±SD) of women and men were 53.5 (±6.8) and 53.4 (±7.3) years (p = 0.858), respectively. The overall prevalence rates (95% confidence intervals (CI)) of insulin resistance, MetS and NAFLD were 59.4% (56.8–62.0%), 62.1% (59.5–64.7%) and 59.3% (56.7–61.9%), respectively.

### Clinical characteristics stratified by insulin resistance, MetS and NAFLD

Differences in demographics, life style habits, and clinical characteristics of subjects stratified by insulin resistance, MetS and NAFLD are presented in Table 1. Generally, when compared with controls, subjects with insulin resistance, MetS and NAFLD had significantly higher levels of waist circumference, systolic and diastolic blood pressure, triglyceride, total cholesterol, fasting plasma glucose (FPG), fasting insulin, glycosylated hemoglobin (HbA1c) and HOMA-IR, and significantly lower level of HDL-C. As for difference of serum uric acid, compared to those controls, subjects with insulin resistance, MetS and NAFLD showed significantly increased SUA levels (350.0 ± 89.8 v.s. 371.9 ± 94.9 μmol/L; 338.8 ± 87.8 v.s. 377.3 ± 93.8 μmol/L and 333.3 ± 84.9 v.s. 383.4 ± 93.7 μmol/L, respectively; all p-values < 0.001) and higher prevalence rates of hyperuricemia (32.9% v.s. 42.4%, 28.8% v.s. 44.5% and 25.7% v.s. 47.3%, respectively; all p-values < 0.001). Subjects with MetS and NAFLD were more likely to be male gender, older and ever-smoker than their controls. There is no significant difference of educational level, ever-drinking and regular physical exercise between subjects with insulin resistance, MetS and NAFLD and those without.

### Clinical characteristics stratified by quartiles of serum uric acid

Table 2 shows the differences in demographics, life style habits, and clinical characteristics stratified by quartiles of SUA. With increasing quartiles of SUA, subjects were more likely to be male gender, ever-smoker, ever-drinker, and had significantly increased levels of waist circumference, systolic and diastolic blood pressure, triglyceride, total cholesterol, LDL-C, fasting insulin, HOMA-IR and decreased level of HDL-C. Increased quartiles of SUA were also significantly associated with increased prevalence rates of insulin resistance, MetS and NAFLD. There was no significant difference in levels of FPG and HbA1C with increasing quartiles of SUA.

### Associations of serum uric acid with insulin resistance, MetS and NAFLD

Adjusted odds ratios (ORs) with associated 95% confidence interval (CI) of SUA for insulin resistance, MetS and NAFLD are shown in Table 3. In model 1, model 2 and model 3 with adjustments for different potential confounders, HUA was significantly associated with increased risks of insulin resistance, MetS and NAFLD. Table 3 also shows that SUA level is significantly associated with increased risks of insulin resistance, MetS and NAFLD, with the adjusted OR (95%CI) of per SD of SUA of 1.231 (1.067–1.420, p = 0.004), 1.345 (1.160–1.560, p < 0.001) and 1.762 (1.528–2.031, p < 0.001), respectively (Model 3). Trend tests for associations of quartiles of SUA with insulin resistance, MetS and NAFLD were all statistically significant (all p-values < 0.001).

To examine if serum uric acid is non-linearly associated with insulin resistance, MetS and NAFLD, logistic regression analyses using multivariable fractional polynomial (MFP) modeling were further conducted. The best FP transformation models show that SUA levels were all linearly and significantly associated with risks of insulin resistance, MetS and NAFLD, with the adjusted OR (95%CI) of per SD of SUA of 1.230 (1.067–1.417, p = 0.004), 1.454 (1.278–1.653, p < 0.001) and 1.383 (1.198–1.595, p < 0.001), respectively (Table 4).

### Possible pathways for serum uric acid leading to NAFLD

**Figure 1:**
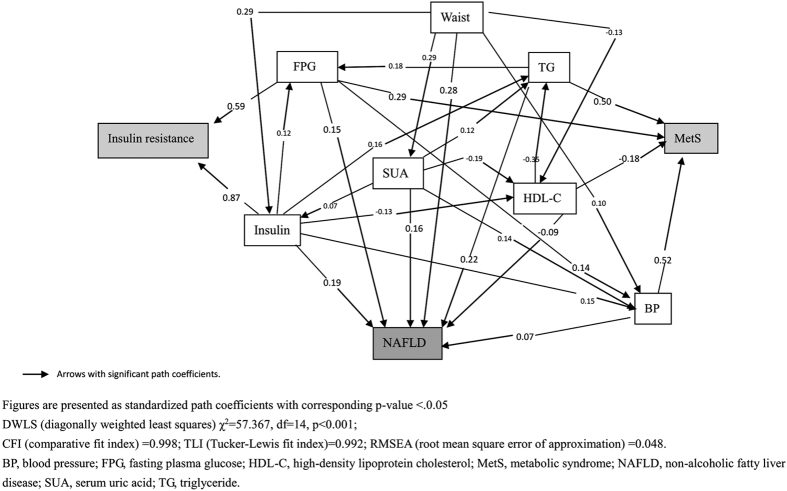
Structural equation modeling path diagrams for associations among serum uric acid, insulin resistance, components of metabolic syndrome with NAFLD.

SEM analysis about the relationships among serum uric acid, insulin resistance, MetS components and NAFLD shows the one-pathway model fits well (χ^2^ = 57.367, p < 0.001; CFI = 0.998; TLI = 0.992; and RMSEA = 0.048) (Fig. 1). The standardized path coefficients in the path diagrams indicate that increased serum uric acid levels might increase fasting insulin, blood pressure, triglyceride and decrease HDL-C, with the corresponding standardized regression coefficients of 0.07, 0.14, 0.12 and −0.19 (all p-values < 0.05), which in turn induce NAFLD. Furthermore, Fig. 1 also shows that increased SUA levels might increase the risk of NAFLD directly, with the standardized path coefficient of 0.16 (p < 0.05).

## Discussion

In this study, we found that increased SUA level was significantly associated with increased prevalence rates of insulin resistance, MetS and NAFLD. After adjustment for potential confounders, increased SUA was significantly associated with increased risk of NAFLD, which is independent of insulin resistance and components of MetS. Furthermore, by using multivariable fractional polynomial modeling, we found from the best FP transformation models that the independent relationship between SUA level and risk of NAFLD is linearly, even for values of SUA within the normal range. Our novel founding on possible pathways of NAFLD by using SEM analysis showed that SUA might induce NAFLD directly besides of the indirect effects through increasing fasting insulin, blood pressure, triglyceride and decreasing HDL-C levels.

NAFLD has been shown to becoming an increasing clinical burden not only confined to liver-related morbidity and mortality but also for extra-hepatic chronic complications. Although population-based estimation of NAFLD prevalence rate is about 30–40% in men and 15–20% in women^17^, the corresponding values for healthy looking obese adults are unknown. In China with the pandemic of obesity due to westernization of lifestyles, the prevalence of NAFLD has increased rapidly for recent decades^18^. In the present study of community-living obese Chinese adults without any previously diagnosed disease, the prevalence rates of NAFLD were about 71.5% for men and 53.8% for women, which highlighted a critical high prevalence for those obese who look healthy. Therefore, screening for NAFLD in the general obese adults should attract attention.

SUA is the end oxidation product of purine metabolism by the liver. Since increased SUA has been consistently shown to be associated with insulin resistance, components of MetS, CVD and renal diseases^5^,^6^,^19^ and all these conditions are also independently associated with NAFLD, interests to explore the association between SUA and NAFLD have grown. Lonardo *et al*. firstly reported a positive relationship between SUA and NAFLD in a case-control study including only 60 Italian NAFLD patients and 60 age- and gender- matched controls^20^. In eastern Asia, Li *et al*. found that HUA was associated with an increased risk of NAFLD in a cross-sectional study including 8925 subjects^7^. However, whether SUA is causal or a consequence of NAFLD remains unclear. A prospective study of 6890 initially NAFLD-free subjects of Li’s study with 3 years’ follow-up, they further found that elevation of SUA could be used to predict incidence of NAFLD^8^. Retrospective evidence was also provided in Korea that HUA was associated with development of NAFLD^9^. In a recent study including both cross-sectional and longitudinal analyses from China, Wu *et al*. found that elevated SUA was associated with risk of NAFLD^21^. For the US populations, studies based on NHANES III (1988–1994) found that HUA or increased quartiles of SUA was associated with increased risk of NAFLD^10^,^11^. Although insulin resistance and components of MetS are critical confounding factors in the relationship between SUA and NAFLD, both of them have not been adjusted for properly in studies above. In the present study of 1365 obese Chinese adults, even though they had relatively higher levels of SUA than general adults, we found that elevated HUA levels were associated with increased risk of NAFLD, and the association remained statistically significant after adjustment for insulin resistance and components of MetS simultaneously.

The “two-hit” hypothesis has been widely accepted for the development of NAFLD^22^. HUA and production of free radicals and inflammatory mediators play important roles in the two hits. MetS and its related components, such as hypertension and T2DM, have been shown to be associated with NAFLD, in which of mechanisms insulin resistance plays a central etiology role of MetS and promotes NAFLD^23^. Evidence from others together with us have consistently shown that SUA is a risk factor or even causal factor of NAFLD^24^, but the mechanisms underlying the effect should be explored further. Apart from the indirect effects of SUA on insulin resistance and components of MetS, which may in turn induce NAFLD, whether there are other pathways about the effect of SUA on NAFLD needs to be clarified. To the best of our knowledge, we are probably the first to examine the possible pathways about the relationships among SUA, insulin resistance, components of MetS and NAFLD, and we found that increased SUA levels might increase fasting insulin, blood pressure and triglyceride and decrease HDL-C levels, which may in turn induce the risk of NAFLD. Besides of that, we also found that SUA might induce NAFLD directly.

SUA can scavenge oxygen radicals and protect the erythrocyte membrane from lipid oxidation. Therefore, it is worthy of exploring if there is a non-linear relationship between SUA level and risk of NAFLD, or whether SUA within normal range is not associated with increased risk of NAFLD. All the available evidence on association between SUA and NAFLD regarded SUA as either continuous variable by assuming a linear relationship between them or categorical variable (quartiles, quintiles or dichotomization into HUA), and this modeling strategy has been criticized often^12^. By using the MFP modeling proposed by Sauerbrei *et al*. in the present study^13^,^14^, we confirmed that SUA levels were linearly associated with increased risk of NAFLD, even for values of SUA within the normal range. Therefore, for obese adults, even those with normal SUA values, screening NAFLD should attract attention, and hypouricemic treatment aimed to prevent NAFLD should be considered if the effect of hypouricemic treatment on NAFLD in humans has been approved.

We should be cautious during interpretation of our findings due to the following limitations. First, subjects were not randomly sampled from their living communities and were all central obese adults and they may have relatively higher SUA than general adults, so we might under-estimate the association between SUA and NAFLD, and our results should be confirmed in non-obese adults. Second, we are not certain about the temporal sequence between SUA and NAFLD because of the cross-sectional study design. Third, we did not measure inflammatory cytokines, such as IL-6 and TNF-α, which may be associated with NAFLD. But we believed this will not eliminate the effect of SUA on NAFLD, since we have adjusted for all components of MetS, which are also associated with inflammatory cytokines, and therefore we have at least partially adjusted for inflammation in the multivariable regression analyses. Another limitation of the present study was that NAFLD was evaluated by hepatic ultrasonography scanning and quantitative sonographic scoring for the degree of hepatic steatosis was not available. In future, we should also noninvasively assess the severity of liver fibrosis and evaluate if SUA levels and hyperuricemia were independent risk factors for liver damage. Last but not the least, dietary fructose intake is a major determinant of SUA, which is also a risk factor for the development and progression of NAFLD and therefore may confound the relationship between SUA and NAFLD. Unfortunately, since the present study did not have data on dietary factors, we cannot exclude the possibility that the association between SUA and NAFLD is accounted for by fructose intake. On the other hand, the present study has a lot of strengths. Our study has a relatively large sample size of obese adults. And we have also adjusted for insulin resistance and components of MetS, which have not been adjusted for properly in previous studies. We are probably the first to explore the potential non-linear relationship and possible pathways between SUA and NAFLD by using the MFP and SEM modeling, respectively.

In summary, the present study demonstrated that prevalence rate of NAFLD in obese adults was much higher than that in the general adults, elevated SUA levels were independently and linearly associated with increased risk of NAFLD. Furthermore, SUA might increase the risk of NAFLD directly besides of the indirect effects through increasing fasting insulin, blood pressure, triglyceride and decreasing HDL-C levels. Our results highlight the urgent need of screening NAFLD and hypouricemic treatment for obese adults.

## Subjects and Methods

### Subjects

**Figure 2:**
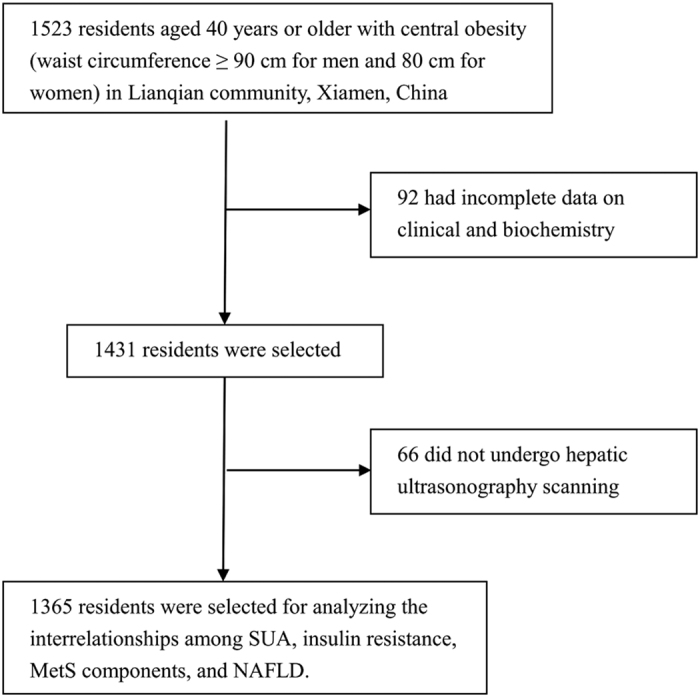
Study subjects selection diagram.

The present study was based on the baseline examination of the same cohort of 1,523 community-living healthy obese Chinese adults as our previous publications^15^,^16^,^25^. Details on methods of subject sampling and recruitment have been described previously^15^,^16^,^25^. Briefly, a total of 1,523 subjects aged 40 years or older living in the Lianqian community, Xiamen, China with central obesity (waist circumference greater than 90 cm for men and 80 cm for women) were recruited from April 2011 to August 2011^15^,^16^,^25^. Of them, 944 females and 421males with the complete information were left for analysis (Fig. 2). The study was approved by the Human Research Ethics Committee of the First Affiliated Hospital of Xiamen University (Xiamen, China) and conducted in compliance with Good Clinical Practice (GCP) guidelines and the Declaration of Helsinki. Written informed consent was obtained from each participant.

### Measurements

Details on clinical and biochemical measurements have been described previously^15^,^16^. After removal of shoes and heavy clothing, each subject underwent weight, height and waist circumference measurements by using a calibrated scale. Body mass index (BMI) was calculated as weight in kilograms divided by height in squared meters as a measure of general obesity. Waist circumference was measured at the midpoint between the inferior costal margin and the superior border of the iliac crest on the midaxillary line^15^,^16^. Body fat were quantified with the Hologic whole body DXA systems (Hologic Inc., Bedford, MA). Arterial blood pressure was measured with a mercury sphygmomanometer after sitting for at least 15 minutes. Blood pressure measurements were taken according to the Joint National Committee VII criteria (JNC VII)^26^.

### Biochemical measurements

All blood samples were obtained after 12-hour fasting. Blood and urine samples were tested in the central laboratory of the First Affiliated Hospital, Xiamen University as described in details previously^15^,^16^. Plasma glucose and serum lipid profiles were determined on a HITACHI 7450 analyzer (HITACHI, Tokyo, Japan). Fasting plasma glucose concentration (FPG) was measured by the hexokinase method. Serum fasting insulin concentration was measured by electrochemiluminiscence immunoassay (Roche Elecsys Insulin Test, Roche Diagnostics, Mannheim, Germany). Homeostasis model assessment - insulin resistance (HOMA-IR) was calculated using the formula: fasting serum insulin (mU/L) *fasting plasma glucose (mmol/L)/22.5. Serum creatinine (Scr) and uric acid were measured by the autoanalyser (COBAS INTEGRA 400 plus, Roche, Basel, Switzerland)^15^,^16^.

HUA was defined as the serum uric acid level >7.0 mg/dL in males and >6.0 mg/dL in females^27^. Insulin resistance was defined as HOMA-IR ≥ 2.6*10^−6^ mol*U/L^2^ 28. MetS was defined by using International Diabetes Federation (IDF) definitions^29^ as described in details previously^15^,^16^.

### Ultrasonography and definition of nonalcoholic fatty liver disease

Hepatic ultrasonography scanning was performed on all subjects by one experienced radiologist using GE LOGIQ P5 scanner (GE Healthcare, Milwaukee, USA) with a 4-MHz probe, who was blinded to the subjects’ health status. Hepatic steatosis was diagnosed on the basis of characteristic sonographic features, including hepatorenal echo contrast, liver parenchymal brightness, deep beam attenuation, and vessel blurring^30^. Quantitative sonographic scoring for the degree of hepatic steatosis was not available in the present study. The definition of NAFLD was based on hepatic ultrasonography diagnosis of hepatic steatosis without excessive alcohol consumption, viral or autoimmune liver disease.

### Statistical analysis

Data were presented as the mean ± standard deviation for continuous variables or number and percentage for categorical variables. Differences between subjects were analyzed using one way ANOVA for continuous variables and chi-square test for categorical variables. Multivariable logistic regression was used to calculate adjusted odds ratio (OR) and 95% confidence intervals (CI) of serum uric acid for insulin resistance, MetS and NAFLD in different models with adjustment for potential confounders. In model 1, age and sex were adjusted for. In model 2, educational level, smoking and drinking habits and regular physical exercise plus model 1 were adjusted for. In model 3, for insulin resistance, adjustment was further made for waist, systolic blood pressure, triglyceride, and HDL-cholesterol; for metabolic syndrome, adjustment was further made for fasting insulin; for NAFLD, adjustment was further made for waist, systolic blood pressure, triglyceride, HDL-cholesterol, fasting plasma glucose and fasting insulin. In each model, SUA was presented separately as HUA (yes v.s. no), per standard deviation (SD) increase of SUA, and quartiles (Q2, Q3, Q4 v.s. Q1).

To examine the non-linear associations for all continuous variables with insulin resistance, MetS and NAFLD, we conducted logistic regression analyses using MFP modeling. The MFP modeling was propose by Sauerbrei *et al*. and applied as a systematic approach to investigate possible non-linear relationships based on fractional polynomials and the combination of linear and non-linear independent variables^12^,^13^,^31^. First- and second-degree FP transformations were considered using a selection level of 0.05 for input variables based on power values of the polynomial ranging (−2, −1, −0.5, 0 (log), 0.5, 1, 2, 3). The best FP for each variable was selected if it resulted in a significantly smaller AIC. The most appropriate transformation was applied to each variable and all variables were considered multivariately based on a backward selection method using a significance level of 0.05 for elimination in each model.

Structural equation modeling (SEM) is a series of statistical methods that allow complex relationships between one or more independent variables and one or more dependent variables. One of the major applications of SEM is causal modeling, which hypothesizes causal relationships among variables and tests a proposed causal process and/or model. In the present study, SEM was used to examine the relationships among serum uric acid, insulin resistance, MetS components, and NAFLD. Standardized path coefficients and the significance of the direct and indirect effects are presented. The path graph (Fig. 1) shows the arrows that represent significant (p-value < 0.05) results. In conducting SEM analysis, satisfactory model fit was indicated by root mean square error of approximation (RMSEA) ≤0.10^32^ and by comparative fit index (CFI) and Tucker-Lewis fit index (TLI) ≥0.90^33^.

SEM was conducted using *lavaan (0.5–19)* package in R3.2.2^34^; MFP and all the other analyses were performed using Stata14.0 (StatCorp, College Station, TX). All p-values were two-sided and p-value < 0.05 was considered statistically significant.

## Additional Information

**How to cite this article:** Liu, C.-Q. *et al*. Serum uric acid is independently and linearly associated with risk of nonalcoholic fatty liver disease in obese Chinese adults. *Sci. Rep.* **6**, 38605; doi: 10.1038/srep38605 (2016).

**Publisher's note:** Springer Nature remains neutral with regard to jurisdictional claims in published maps and institutional affiliations.

## Figures and Tables

**Table 1:** Demographic, lifestyle and clinical characteristics of subjects by insulin resistance, metabolic syndrome and NAFLD.

Variables	Insulin resistance (IR)			Metabolic syndrome (MetS)			NAFLD		
	No	Yes	P value	No	Yes	P value	No	Yes	P value
**Demographics**									
N (%)	554 (40.6%)	811 (59.4%)		517 (37.9%)	848 (62.1%)		556 (40.7%)	809 (59.3%)	
Sex			0.276			<0.001‡			<0.001‡
Female (n, %)	374 (67.5%)	570 (70.3%)		386 (74.7%)	558 (65.8%)		436 (78.4%)	508 (62.8%)	
Male (n, %)	180 (32.5%)	241 (29.7%)		131 (25.3%)	290 (34.2%)		120 (21.6%)	301 (37.2%)	
Age (years)	53.0 ± 7.0	53.7 ± 7.0	0.092	52.1 ± 6.8	54.3 ± 7.0	<0.001‡	52.7 ± 6.9	53.9 ± 7.0	0.002†
Education, (n, %)			0.516			0.362			0.163
Illiteracy	143 (25.8%)	234 (28.9%)		141 (27.3%)	236 (27.8%)		166 (29.9%)	211 (26.1%)	
Elementary school	164 (29.6%)	236 (29.1%)		156 (30.2%)	244 (28.8%)		173 (31.1%)	227 (28.1%)	
Middle school	131 (23.7%)	185 (22.8%)		113 (21.9%)	203 (23.9%)		119 (21.4%)	197 (24.4%)	
High school	72 (13.0%)	108 (13.3%)		64 (12.4%)	116 (13.7%)		64 (11.5%)	116 (14.3%)	
College or above	44 (7.9%)	48 (5.9%)		43 (8.3%)	49 (5.8%)		34 (6.1%)	58 (7.2%)	
**Life style**									
Ever smoking (n, %)	158 (28.5%)	204 (25.2%)	0.167	121 (23.4%)	241 (28.4%)	0.042*	113 (20.3%)	249 (30.8%)	<0.001‡
Ever drinking (n, %)	92 (16.6%)	120 (14.8%)	0.365	71 (13.7%)	141 (16.6%)	0.152	73 (13.1%)	129 (15.9%)	0.173
Regular physical exercise (n, %)	197 (35.6%)	249 (30.7%)	0.060	162 (31.3%)	284 (33.5%)	0.410	193 (34.7%)	253 (31.3%)	0.183
**Clinical characteristics**									
Waist circumference (cm)	91.8 ± 6.0	94.8 ± 7.5	<0.001‡	92.1 ± 6.4	94.5 ± 7.2	<0.001‡	90.8 ± 5.6	95.5 ± 7.3	<0.001‡
Systolic blood pressure (mmHg)	128.9 ± 16.3	136.8 ± 17.5	<0.001‡	122.8 ± 13.7	140.1 ± 16.3	<0.001‡	129.4 ± 17.1	136.5 ± 17.2	<0.001‡
Diastolic blood pressure (mmHg)	77.1 ± 10.0	81.3 ± 10.8	<0.001‡	73.9 ± 8.6	83.1 ± 10.3	<0.001‡	76.9 ± 10.2	81.5 ± 10.6	<0.001‡
Triglyceride (mmol/L)	1.52 ± 0.95	2.11 ± 1.44	<0.001‡	1.22 ± 0.63	2.27 ± 1.43	<0.001‡	1.41 ± 0.99	2.19 ± 1.38	<0.001‡
Total cholesterol (mmol/L)	5.79 ± 1.02	5.93 ± 1.14	0.017*	5.73 ± 0.99	5.96 ± 1.14	<0.001‡	5.72 ± 1.06	5.98 ± 1.10	<0.001‡
HDL-cholesterol (mmol/L)	1.43 ± 0.30	1.33 ± 0.29	<0.001‡	1.51 ± 0.29	1.29 ± 0.27	<0.001‡	1.47 ± 0.31	1.31 ± 0.27	<0.001‡
LDL-cholesterol (mmol/L)	3.67 ± 0.92	3.65 ± 1.06	0.646	3.67 ± 0.91	3.65 ± 1.06	0.734	3.61 ± 0.95	3.69 ± 1.04	0.134
Fasting glucose (mmol/L)	5.54 ± 0.59	6.55 ± 2.05	<0.001‡	5.55 ± 1.08	6.49 ± 1.90	<0.001‡	5.80 ± 1.06	6.37 ± 1.99	<0.001‡
Fasting insulin (mU/L)	7.6 ± 1.9	16.1 ± 7.0	<0.001‡	10.0 ± 4.6	14.3 ± 7.7	<0.001‡	10.2 ± 5.5	14.4 ± 7.3	<0.001‡
HOMA-IR (*10^−6^mol*IU*L^−2^)	1.86 ± 0.47	4.67 ± 2.81	<0.001‡	2.50 ± 1.29	4.15 ± 2.95	<0.001‡	2.70 ± 2.34	4.10 ± 2.59	<0.001‡
HbA1c	5.90 ± 0.45	6.40 ± 1.23	<0.001‡	5.91 ± 0.60	6.38 ± 1.17	<0.001‡	5.95 ± 0.66	6.37 ± 1.18	<0.001‡
Hyperuricemia, (n (%))	182 (32.9%)	344 (42.4%)	<0.001‡	149 (28.8%)	377 (44.5%)	<0.001‡	143 (25.7%)	383 (47.3%)	<0.001‡
Serum uric acid (mmol/L)	350.0 ± 89.8	371.9 ± 94.9	<0.001‡	338.8 ± 87.8	377.3 ± 93.8	<0.001‡	333.3 ± 84.9	383.4 ± 93.7	<0.001‡

^*^p < 0.05, ^†^p < 0.01, ^‡^p < 0.001.

All percentages are column percentage; except for percentages, all values are mean ± SD.

Abbreviations: HDL, high-density lipoprotein; HOMA, homeostasis model assessment; IR, insulin resistance index; LDL, low-density lipoprotein cholesterol; NAFLD, non-alcoholic fatty liver disease.

**Table 2:** Demographic, lifestyle and clinical characteristics of subjects by quartiles of serum uric acid.

Variables	Quartile 1	Quartile 2	Quartile 3	Quartile 4	P value
**Demographics**					
N (%)	341 (25.0%)	341 (25.0%)	343 (25.1%)	340 (24.9%)	
Sex					<0.001‡
Female (n, %)	320 (93.8%)	295 (86.5%)	209 (60.9%)	120 (35.3%)	
Male (n, %)	21 (6.2%)	46 (13.5%)	134 (39.1%)	220 (64.7%)	
Age (years)	52.9 ± 7.2	54.0 ± 6.6	53.4 ± 7.0	53.5 ± 7.1	0.230
Education, (n, %)					0.019*
Illiteracy	104 (30.5%)	109 (32.0%)	87 (25.4%)	77 (22.7%)	
Elementary school	83 (24.3%)	102 (29.9%)	106 (30.9%)	109 (32.1%)	
Middle school	74 (21.7%)	73 (21.4%)	85 (24.8%)	84 (24.7%)	
High school	51 (15.0%)	46 (13.5%)	43 (12.5%)	40 (11.8%)	
College or above	29 (8.5%)	11 (3.2%)	22 (6.4%)	30 (8.8%)	
**Life style**					
Ever smoking (n, %)	23 (6.7%)	46 (13.5%)	113 (32.9%)	180 (52.9%)	<0.001‡
Ever drinking (n, %)	15 (4.4%)	25 (7.3%)	66 (19.2%)	106 (31.2%)	<0.001‡
Regular physical exercise (n, %)	107 (31.4%)	111 (32.6%)	114 (33.2%)	114 (33.5%)	0.935
**Clinical characteristics**					
Waist circumference (cm)	91.4 ± 6.4	92.7 ± 6.7	94.3 ± 6.8	95.9 ± 7.4	<0.001‡
Systolic blood pressure (mmHg)	129.8 ± 17.7	132.2 ± 17.8	135.1 ± 17.3	137.3 ± 16.3	<0.001‡
Diastolic blood pressure (mmHg)	76.9 ± 10.1	78.3 ± 10.6	81.2 ± 10.6	82.0 ± 10.6	<0.001‡
Triglyceride (mmol/L)	1.52 ± 1.11	1.77 ± 1.22	1.96 ± 1.24	2.23 ± 1.49	<0.001‡
Total cholesterol (mmol/L)	5.70 ± 1.02	5.90 ± 1.10	5.98 ± 1.17	5.92 ± 1.05	0.006†
HDL-cholesterol (mmol/L)	1.48 ± 0.31	1.40 ± 0.29	1.33 ± 0.28	1.29 ± 0.28	<0.001‡
LDL-cholesterol (mmol/L)	3.54 ± 0.94	3.71 ± 0.98	3.76 ± 1.06	3.62 ± 1.03	0.021*
Fasting glucose (mmol/L)	6.18 ± 2.19	6.17 ± 1.89	6.18 ± 1.52	6.01 ± 0.93	0.493
Fasting insulin (mU/L)	10.8 ± 4.7	12.7 ± 7.2	13.3 ± 7.6	13.8 ± 7.6	<0.001‡
HOMA-IR (*10^−6^mol*IU*L−^2^)	2.97 ± 1.68	3.60 ± 2.80	3.77 ± 3.18	3.77 ± 2.38	<0.001‡
HbA1c	6.20 ± 1.22	6.26 ± 1.17	6.22 ± 0.99	6.13 ± 0.57	0.398
Serum uric acid (mmol/L)	253.4 ± 32.8	324.3 ± 16.0	386.4 ± 19.5	488.0 ± 60.4	<0.001‡
Insulin resistance, (n, %)	173 (50.7%)	199 (58.4%)	219 (63.9%)	220 (64.7%)	0.001†
Metabolic syndrome, (n, %)	166 (48.7%)	201 (58.9%)	235 (68.5%)	246 (72.4%)	<0.001‡
NAFLD, (n, %)	145 (42.5%)	177 (51.9%)	231 (67.4%)	256 (75.3%)	<0.001‡

^*^p < 0.05, ^†^p < 0.01, ^‡^p < 0.001.

All percentages are column percentage; except for percentages, all values are mean ± SD; Abbreviations: HDL, high-density lipoprotein; HOMA, homeostasis model assessment; IR, insulin resistance index; LDL, low-density lipoprotein cholesterol; NAFLD, non-alcoholic fatty liver disease.

**Table 3:** Adjusted odds ratios (ORs) with associated 95% confidence interval (CI) for insulin resistance, metabolic syndrome and NAFLD.

Variables	Insulin resistance			Metabolic syndrome			NAFLD		
	OR	95%CI	P value	OR	95%CI	P value	OR	95%CI	P value
**Model 1**									
Hyperuricemia (yes v.s. no)	1.567	1.245–1.974	<0.001*	1.890	1.486–2.404	<0.001*	2.370	1.864–3.012	<0.001*
Serum uric acid†	1.434	1.257–1.636	<0.001*	1.581	1.374–1.818	<0.001*	1.722	1.496–1.983	<0.001*
Serum uric acid ^‡^									
(Quartile 2 vs. Quartile 1)	1.393	1.027–1.889	0.033*	1.447	1.064–1.968	0.018*	1.396	1.029–1.893	0.032*
(Quartile 3 vs. Quartile 1)	2.012	1.456–2.780	<0.001*	2.256	1.623–3.135	<0.001*	2.552	1.844–3.533	<0.001*
(Quartile 4 vs. Quartile 1)	2.363	1.658–3.367	<0.001*	2.685	1.864–3.866	<0.001*	3.524	2.445–5.079	<0.001*
Trend test			<0.001*			<0.001*			<0.001*
**Model 2**									
Hyperuricemia (yes v.s. no)	1.565	1.242–1.973	<0.001*	1.884	1.480–2.396	<0.001*	2.423	1.903–3.086	<0.001*
Serum uric acid†	1.436	1.258–1.639	<0.001*	1.582	1.375–1.821	<0.001*	1.762	1.528–2.031	<0.001*
Serum uric acid‡									
(Quartile 2 vs. Quartile 1)	1.390	1.024–1.888	0.035*	1.441	1.059–1.962	0.020*	1.440	1.059–1.958	0.020*
(Quartile 3 vs. Quartile 1)	2.011	1.453–2.781	<0.001*	2.247	1.616–3.126	<0.001*	2.648	1.908–3.676	<0.001*
(Quartile 4 vs. Quartile 1)	2.359	1.652–3.367	<0.001*	2.678	1.856–3.862	<0.001*	3.715	2.566–5.378	<0.001*
Trend test			<0.001*			<0.001*			<0.001*
**Model 3**									
Hyperuricemia (yes v.s. no)	1.268	0.987–1.630	0.063	1.533	1.186–1.982	0.001*	1.992	1.516–2.618	<0.001*
Serum uric acid†	1.231	1.067–1.420	0.004*	1.345	1.160–1.560	<0.001*	1.762	1.528–2.031	<0.001*
Serum uric acid ^‡^									
(Quartile 2 vs. Quartile 1)	1.176	0.846–1.634	0.335	1.163	0.839–1.613	0.365	1.105	0.779–1.567	0.575
(Quartile 3 vs. Quartile 1)	1.545	1.091–2.188	0.014*	1.652	1.165–2.342	0.005*	1.908	1.318–2.762	0.001*
(Quartile 4 vs. Quartile 1)	1.678	1.142–2.466	0.008*	1.748	1.180–2.588	0.005*	2.475	1.622–3.777	<0.001*
Trend test			0.004*			0.001*			<0.001*

^*^p < 0.05.

^†^OR and 95%CI was impressed by per SD increase of serum uric acid.

^‡^OR and 95%CI was impressed by the first quartile of serum uric acid as the reference.

Model 1: was adjusted for sex and age. Model 2: was further adjusted for educational level, ever smoking, ever drinking and regular physical exercise. Model 3: For insulin resistance, model 3 was further adjusted for waist, systolic blood pressure, triglyceride, HDL-cholesterol; For metabolic syndrome, model 3 was further adjusted for fasting insulin; For NAFLD, model 3 was further adjusted for waist, systolic blood pressure, triglyceride, HDL-cholesterol, fasting plasma glucose and fasting insulin.

**Table 4:** Adjusted odds ratios (ORs) with associated 95% confidence interval (CI) for insulin resistance, metabolic syndrome and NAFLD using multivariable fractional polynomial modelling logistic regression.

Variables	In/Out of final model	MFP transformation			OR (95%CI)	
		FP	Transformation	P value	OR	95%CI
Insulin Resistance						
**Categorical**						
Sex (male v.s. female)	In	N/A		<0.001*	0.315	0.228–0.435
Education	Out					
Ever smoking	Out					
Ever drinking	Out					
Regular physical exercise	Out					
**Continuous**						
Age (years)	Out					
Waist circumference (cm)	In	Linear		<0.001*	1.071	1.049–1.093
Systolic blood pressure (mmHg)	In	Linear		<0.001*	1.024	1.017–1.032
Triglyceride (mmol/L)	In	Linear		<0.001*	1.407	1.242–1.595
HDL-cholesterol (mmol/L)	In	Linear		0.003*	0.505	0.319–0.798
Fasting glucose (mmol/L)	N/A					
Fasting insulin (mU/L)	N/A					
Serum uric acid (μmol/L)†	In	Linear		0.004*	1.230	1.067–1.417
**Metabolic Syndrome**						
**Categorical**						
Sex (male v.s. female)	Out					
Education	Out					
Ever smoking	Out					
Ever drinking	Out					
Regular physical exercise	Out					
**Continuous**						
Age (years)	In	Linear		<0.001*	1.053	1.035–1.072
Waist circumference (cm)	N/A					
Systolic blood pressure (mmHg)	N/A					
Triglyceride (mmol/L)	N/A					
HDL-cholesterol (mmol/L)	N/A					
Fasting glucose (mmol/L)	N/A					
Fasting insulin (mU/L)	In	FP1(0)	log (fasting insulin)	<0.001*	5.456	4.077–7.301
Serum uric acid (μmol/L)†	In	Linear		<0.001*	1.454	1.278–1.653
**NAFLD**						
**Categorical**						
Sex (male v.s. female)	Out					
Education	Out					
Ever smoking	Out					
Ever drinking	Out					
Regular physical exercise	Out					
**Continuous**						
Age (years)	Out					
Waist circumference (cm)	In	Linear		<0.001*	1.093	1.068–1.118
Systolic blood pressure (mmHg)	Out					
Triglyceride (mmol/L)	In	FP1 (−0.5)	triglyceride^−0.5^	<0.001*	0.408	0.335–0.498
HDL-cholesterol (mmol/L)	Out					
Fasting glucose (mmol/L)	In	Linear		0.001*	1.176	1.068–1.295
Fasting insulin (mU/L)	In	FP2 (0.5, 3)	insulin^0.5^	<0.001*	11.473	6.020–21.867
			insulin^0.5^*insulin^3^	0.007*	0.987	0.978–0.996
Serum uric acid (μmol/L)†	In	Linear		<0.001*	1.383	1.198–1.595

^*^p < 0.05.

^†^OR and 95%CI was impressed by per SD increase of serum uric acid.

## References

[b1] Angulo P. Nonalcoholic fatty liver disease. N Engl J Med. 346, 1221–1231 (2002). 11961152 10.1056/NEJMra011775

[b2] Masarone M., Federico A., Abenavoli L., Loguercio C. & Persico M. Non alcoholic fatty liver: epidemiology and natural history. Rev Recent Clin Trials. 9, 126–133 (2014). 25514916 10.2174/1574887109666141216111143

[b3] Targher G. & Byrne C. D. Clinical Review: Nonalcoholic fatty liver disease: a novel cardiometabolic risk factor for type 2 diabetes and its complications. J Clin Endocrinol Metab. 98, 483–495 (2013). 23293330 10.1210/jc.2012-3093

[b4] Byrne C. D. & Targher G. NAFLD: a multisystem disease. J Hepatol. 62 (1 Suppl), S47–64 (2015). 25920090 10.1016/j.jhep.2014.12.012

[b5] Richette P. & Perez-Ruiz F. Serum uric acid and metabolic risk. Curr Med Res Opin. 29 (Suppl 3), 9–15 (2013). 23611367 10.1185/03007995.2013.790801

[b6] Yuan H. . Serum uric acid levels and risk of metabolic syndrome: a dose-response meta-analysis of prospective studies. J Clin Endocrinol Metab. 100, 4198–4207 (2015). 26308292 10.1210/jc.2015-2527

[b7] Li Y., Xu C., Yu C., Xu L. & Miao M. Association of serum uric acid level with non-alcoholic fatty liver disease: a cross-sectional study. J Hepatol. 50, 1029–1034 (2009). 19299029 10.1016/j.jhep.2008.11.021

[b8] Xu C., Yu C., Xu L., Miao M. & Li Y. High serum uric acid increases the risk for nonalcoholic Fatty liver disease: a prospective observational study. PLoS One. 5, e11578 (2010). 20644649 10.1371/journal.pone.0011578 PMC2904389

[b9] Lee J. W. . Serum uric Acid as a predictor for the development of nonalcoholic Fatty liver disease in apparently healthy subjects: a 5-year retrospective cohort study. Gut Liver. 4, 378–383 (2010). 20981217 10.5009/gnl.2010.4.3.378 PMC2956352

[b10] Shih M. H. . Association between serum uric acid and nonalcoholic fatty liver disease in the US population. J Formos Med Assoc. 114, 314–320 (2015). 25839764 10.1016/j.jfma.2012.11.014 PMC6312686

[b11] Sirota J. C. . Elevated serum uric acid levels are associated with non-alcoholic fatty liver disease independently of metabolic syndrome features in the United States: Liver ultrasound data from the National Health and Nutrition Examination Survey. Metabolism. 62, 392–399 (2013). 23036645 10.1016/j.metabol.2012.08.013 PMC3565047

[b12] Royston P., Altman D. G. & Sauerbrei W. Dichotomizing continuous predictors in multiple regression: a bad idea. Stat Med. 25, 127–141 (2006). 16217841 10.1002/sim.2331

[b13] Royston P., Ambler G. & Sauerbrei W. The use of fractional polynomials to model continuous risk variables in epidemiology. Int J Epidemiol. 28, 964–974 (1999). 10597998 10.1093/ije/28.5.964

[b14] Sauerbrei W., Meier-Hirmer C., Benner A. & Royston P. Multivariable regression models by using fractional polynomials: description of SAS, Stata and R programs. Comput Stat Data Anal. 50, 3463–3485 (2006).

[b15] Yan B. . Association of serum irisin with metabolic syndrome in obese Chinese adults. PLoS One. 9, e94235 (2014). 24709991 10.1371/journal.pone.0094235 PMC3978033

[b16] Yang S. . Association of serum irisin and body composition with chronic kidney disease in Chinese adults: a cross-sectional study. BMC Nephrology. 16, doi: 10.1186/s12882-015-0009-5 (2015). PMC4365765 25884312

[b17] Browning J. D. . Prevalence of hepatic steatosis in an urban population in the United States: impact of ethnicity. Hepatology. 40, 1387–1395 (2004). 15565570 10.1002/hep.20466

[b18] Hou X. H. . Non-alcoholic fatty liver disease’s prevalence and impact on alanine aminotransferase associated with metabolic syndrome in the Chinese. J Gastroenterol Hepatol. 26, 722–730 (2011). 21418302 10.1111/j.1440-1746.2010.06509.x

[b19] Tsouli S. G., Liberopoulos E. N., Mikhailidis D. P., Athyros V. G. & Elisaf M. S. Elevated serum uric acid levels in metabolic syndrome: an active component or an innocent bystander? Metabolism. 55, 1293–1301 (2006). 16979398 10.1016/j.metabol.2006.05.013

[b20] Lonardo A. . Fasting insulin and uric acid levels but not indices of iron metabolism are independent predictors of non-alcoholic fatty liver disease. A case-control study. Dig Liver Dis. 34, 204–211 (2002). 11990393 10.1016/s1590-8658(02)80194-3

[b21] Wu S. J. . Association between sex-specific serum uric acid and non-alcoholic fatty liver disease in Chinese adults: a large population-based study. Medicine (Baltimore). 94, e802 (2015). 25929934 10.1097/MD.0000000000000802 PMC4603030

[b22] Day C. P. & James O. F. Steatohepatitis: a tale of two “hits”? Gastroenterology. 114, 842–845 (1998). 9547102 10.1016/s0016-5085(98)70599-2

[b23] Bugianesi E., McCullough A. J. & Marchesini G. Insulin resistance: a metabolic pathway to chronic liver disease. Hepatology. 42, 987–1000 (2005). 16250043 10.1002/hep.20920

[b24] Sun D. Q. . Serum uric acid: a new therapeutic target for nonalcoholic fatty liver disease. Expert Opin Ther Targets. 20(3), 375–87 (2016). 26419119 10.1517/14728222.2016.1096930

[b25] Zhang H. J. . Irisin is inversely associated with intrahepatic triglyceride contents in obese adults. J Hepatol. 59, 557–562 (2013). 23665283 10.1016/j.jhep.2013.04.030

[b26] Chobanian A. V. . The Seventh Report of the Joint National Committee on Prevention, Detection, Evaluation, and Treatment of High Blood Pressure: the JNC 7 report. JAMA. 289, 2560–2572 (2003). 12748199 10.1001/jama.289.19.2560

[b27] Chuang S. Y., Chen J. H., Yeh W. T., Wu C. C. & Pan W. H. Hyperuricemia and increased risk of ischemic heart disease in a large Chinese cohort. Int J Cardiol. 154, 316–321 (2012). 21862159 10.1016/j.ijcard.2011.06.055

[b28] Ascaso J. F. . Diagnosing insulin resistance by simple quantitative methods in subjects with normal glucose metabolism. Diabetes Care. 26, 3320–3325 (2003). 14633821 10.2337/diacare.26.12.3320

[b29] Alberti K. G. . Harmonizing the metabolic syndrome: a joint interim statement of the International Diabetes Federation Task Force on Epidemiology and Prevention; National Heart, Lung, and Blood Institute; American Heart Association; World Heart Federation; International Atherosclerosis Society; and International Association for the Study of Obesity. Circulation. 120, 1640–1645 (2009). 19805654 10.1161/CIRCULATIONAHA.109.192644

[b30] Jian-gao F. Chinese Liver Disease Association: Guidelines for management of nonalcoholic fatty liver disease. An updated and revised edition. Zhonghua Gan Zang Bing Za Zhi. 18, 163–166, In Chinese (2010). 20698076

[b31] Silke B., Kellett J., Rooney T., Bennett K. & O’Riordan D. An improved medical admissions risk system using multivariable fractional polynomial logistic regression modelling. QJM. 103, 23–32 (2010). 19846579 10.1093/qjmed/hcp149

[b32] Kline R. B. Principles and Practice of Structural Equation Modeling. (New York: The Guilford Press, 1998).

[b33] Bentler P. M. On the fit of models to covariances and methodology to the Bulletin. Psychological Bulletin. 112, 400–404 (1992). 1438635 10.1037/0033-2909.112.3.400

[b34] R Core Team R: A language and environment for statistical computing. R Foundation for Statistical Computing, Vienna, Austria. URL: http://www.R-project.org/ (2015).

